# Immunosuppressive Induction Therapy Using the Antithymocyteglobulin Grafalon: A Single-Center Non-Interventional Study

**DOI:** 10.3390/jcm13144051

**Published:** 2024-07-11

**Authors:** Nikolaus Becker, David Pereyra, Jule Dingfelder, Chiara Tortopis, Tina Saffarian Zadeh, Moriz Riha, Sertac Kacar, Thomas Soliman, Gabriela A. Berlakovich, Georg Györi

**Affiliations:** 1 Department of General Surgery, Division of Transplantation, Medical University of Vienna, 1090 Wien, Austriathomas.soliman@meduniwien.ac.at (T.S.); gabriela.berlakovich@meduniwien.ac.at (G.A.B.); georg.gyoeri@meduniwien.ac.at (G.G.); 2 Department of General Surgery, Division of Visceral Surgery, Medical University of Vienna, 1090 Wien, Austria

**Keywords:** induction therapy, immunosuppression, liver transplantation, real-world evidence, safety, biopsy-proven acute rejection, ATG, Grafalon, Thymoglobulin

## Abstract

**Background:** Induction therapy with depleting antibodies in the setting of liver transplantation (LT) is discussed controversially to this day. The rabbit antithymocyteglobulin (ATG) Thymoglobulin (rATG) was introduced as early as 1984 and was frequently used as a standard regime for induction therapy after LT. There are no public reports characterizing Grafalon (ATG-F), a novel ATG, as an induction agent after LT. **Objectives**: The aim of this observational non-interventional study was to investigate the safety and efficacy of Grafalon induction therapy and characterize its clinical effects in the setting of LT. **Methods**: A cohort of 80 patients undergoing deceased donor LT at the Medical University of Vienna and receiving Grafalon as part of the clinical standard immunosuppressive regimen was prospectively included between March 2021 and November 2022. Patients were monitored closely for leukocytopenia and thrombocytopenia during the first postoperative week and followed up for incidence and severity of biopsy-proven acute rejection (BPAR), overall survival, and bacterial infections in the first year after LT. **Results**: The incidences of thrombocytopenia and leukocytopenia following Grafalon treatment peaked on postoperative day four, with 64% and 31%, respectively. However, there were no cases of severe leukocytopenia after the first postoperative week. Induction therapy with Grafalon resulted in a rate of localized bacterial infections and bacteremia of 28% and 21%, respectively. The rate of BPAR was 12.5% in the first year after LT; the one-year survival rate in this cohort was 90%. **Conclusions**: Overall, this study provides evidence of the safety and efficacy of Grafalon as an induction agent. Further studies investigating the potential long-term effects of Grafalon, as well as comparison studies with different immunosuppressive regimens, are needed in order to draw further conclusions.

## 1. Introduction

Liver transplantation (LT) remains the last resort in the treatment of therapy-resistant acute liver failure and chronic end-stage liver diseases [1,2]. However, with demand increasingly exceeding the supply of donor grafts, improving the efficiency of LT has been at the center of research. Major levers that can be adjusted include ameliorating the allocation process and identifying recipients in urgent need [3,4,5,6], salvaging marginal and treating viable organs before transplantation [7,8,9], and advancing patient care [10,11,12,13,14,15]. For the latter, optimizing immunosuppressive (IS) therapy is an important but controversially discussed element, and different strategies have been pursued.

The main challenge in IS therapy is to establish a balance between over- and undersuppression in order to avoid high susceptibility to infections and malignancy or graft loss and rejection, respectively [15,16,17]. In the last few decades, one-year survival following LT has constantly risen from 66% in 1986 to 92% in 2015. In parallel, long-term survival did not improve comparably, and mortality due to oversuppression remains high. Malignancy and infection as potential side effects of immunosuppression account for 27% of long-term deaths following LT, as opposed to 2% of deaths due to acute rejection [17]. Together with the wide variety of IS regimens that differ from center to center, this illustrates the urgent need for more clinical evidence in post-LT immunosuppression [10,11,17].

In contrast to IS maintenance therapy, mainly consisting of corticosteroids, calcineurin inhibitors (CNIs), or molecular targets of rapamycin (mTOR) inhibitors, induction regimens use T-cell-depleting biological agents like Thymoglobulin, Grafalon, or monoclonal antibodies (e.g., alemtuzumab, anti-CD52). The combination of individual IS drugs is thought to reduce side effects by the low dosing of individual therapeutics whilst maintaining optimal IS and thereby minimizing rejection. While the main molecular mechanism targeted by CNI and mTOR inhibitors is IL2-dependent T-cell activation and proliferation, corticosteroids generally inhibit leukocyte activation and cytokine release [16]. In contrast, polyclonal antibodies like Thymoglobulin or Grafalon have a broad spectrum of cellular targets, with different pathophysiological mechanisms [16,18,19]. While Thymoglobulin and Grafalon are both produced in rabbits and target T-cells, one main difference between these agents is posed by the T-cell lineages used for immunization. In particular, human thymocytes are used for the generation of Thymoglobulin, and the human Jurkat T-cell line is used to produce Grafalon [19]. However, the clinical consequences of the different antibody profiles of Thymoglobulin and Grafalon are not yet understood and there are no published studies comparing the clinical effects of the two most widely used polyclonal antithymocyteglobulin (ATG) formulations in the context of LT.

The aim of this observational, non-interventional, single-center study was to evaluate the safety and efficacy of post-LT induction therapy using the ATG formulation Grafalon.

## 2. Patients and Methods

### 2.1. Study Cohort and Ethical Approval

In this study, we prospectively included 80 patients who underwent de novo deceased donor LT from March 2021 to November 2022 at the Medical University of Vienna. All patients provided informed consent in written form and agreed to participate in the clinical visits. This study was conducted in alignment with the Declaration of Helsinki. Ethical approval was granted by the Institutional Ethics Committee of the Medical University of Vienna (protocol code 2159/2020, 19 November 2020).

### 2.2. Immunosuppressive Regimen

All patients received Grafalon (Neovii Biotech GmbH, Gräfelfing, Germany) induction therapy as part of the standard clinical IS regimen. Grafalon was administered on postoperative days (PODs) one to three; the target dose was 1.5 mg/kg/day, with a daily maximum of 160 mg. In the case of mild leukocytopenia (<3 × 10^3^/L), patients received a 50% dose reduction; in the case of severe leukocytopenia (<2 × 10^3^/L), Grafalon was paused. Patients with leukocytopenia < 1 × 10^3^/L additionally received 48 Mio. IE of filgrastim. Furthermore, high-dose corticosteroids were established intraoperatively (250 mg i.v. prednisolone), followed by a rapid taper scheme (125mg on POD 1, 50 mg POD 2–6; p.o.: 25 mg/day on PODs 7–14, 10 mg/day on PODs 15–29, 5 mg/day afterwards for 3–6 months). Tacrolimus completed local clinical standard immunosuppression; treatment was established on POD 4 and adapted to a blood trough level of 6–8 ng/mL, starting with an initial dose of 0.1 mg/kg.

### 2.3. Patient Monitoring and Follow-Up

The observational period was defined as one year post-LT, in accordance with the guidelines on the clinical investigation of immunosuppressants for solid organ transplantation by the European Medicine Agency (EMA) [20]. Patients’ baseline demographics were prospectively evaluated at the time of listing for transplantation. Type 1 and type 2 diabetes mellitus were classified using the 2024 ADA guidelines [21]. During the first postoperative week, patients were clinically monitored routinely in accordance with local clinical standards. Physical examinations, vital signs, laboratory tests, concomitant drugs, and documentation of adverse drug reactions and unrelated adverse events were assessed at each visit; there were no additional study-mandated medical procedures. A liver biopsy was performed in the case of suspected acute rejection, which was confirmed as defined below. After discharge, patient visits were scheduled at PODs 14 ± 2, 30 ± 3, 90 ± 5, and 365 ± 14. An analysis of the relatedness of reported bacterial infections was performed post hoc.

### 2.4. Study Endpoints and Definitions

Primary efficacy endpoints were the incidence and severity of BPAR, graft failure (i.e., need for re-transplantation), and patient death. In patients with clinical signs of rejection, i.e., an at least ten percent increase in transaminase concentrations on two consecutive days, liver biopsies were evaluated by the pathologist on duty, in accordance with local clinical standards. BPAR was defined as any histological sign of portal inflammation, bile duct damage, or venous endothelial inflammation, and severity was graded using the rejection activity index (RAI), as described in the revised Banff guidelines from 2016 [22]. Tolerability endpoints included the development of bacterial infection (surgical site infection, pneumonia, and positive blood culture), risk of malignancy or post-transplant lymphoproliferative disorders (PTLDs), and, specifically during the first week post-LT, the incidence and severity of leukocytopenia and thrombocytopenia.

### 2.5. Statistical Analysis

All data analysis was performed using R Statistical Software (v4.2.2; R Core Team 2022). The level of significance was defined as *p* < 0.05. Patient characteristics were described using frequencies and numbers or means and standard deviations. Endpoints were described using frequencies and numbers. The potential adverse effects of Grafalon were shown as incidences or medians and ranges. T-cell depletion was statistically investigated using Spearman’s correlation between relative changes in leukocyte counts from POD 1 to POD 7 and total Grafalon dose.

## 3. Results

### 3.1. Patient Characteristics

In this study, we included 80 patients who underwent de novo deceased donor LT from March 2021 to November 2022 at the Medical University of Vienna and who were treated with Grafalon as an induction therapy as part of local standard immunosuppression following LT. Baseline patient demographics and characteristics are visualized in Table 1.

### 3.2. Thrombocytopenia and Leukocytopenia

On average, patients received a total of 366 mg ± 72 mg Grafalon in three doses. Besides clinical observation, SpO2, blood pressure, and ECG during infusion, lab parameters were routinely assessed in the postoperative care to adapt daily doses in cases of leukocytopenia (mild: <3 × 10^3^/L, severe: <2 × 10^3^/L).

There were no acute adverse events associated with the infusion of Grafalon. The incidence of thrombocytopenia peaked on POD 4 affecting 64% (n = 51) of patients, while platelet counts recovered up to POD 7 showed only 29% (n = 23) still affected by thrombocytopenia.

**Figure 1:**
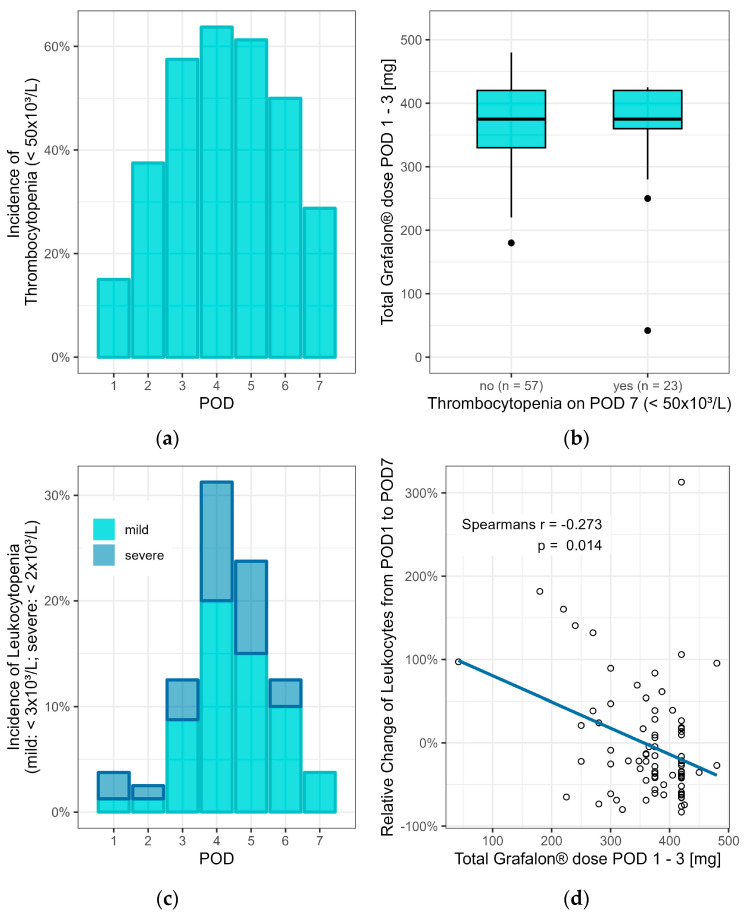
(**a**) The incidence of thrombocytopenia peaked on POD 4. (**b**) The occurrence of thrombocytopenia on POD 7 was not dose-dependent. (**c**) The incidence of leukocytopenia also peaked on POD 4; however, severe leukocytopenia did not persist after POD 7. (**d**) A dose-dependent leukocyte-depleting effect of Grafalon could be observed. POD, postoperative day.

On POD 14, only two patients displayed platelet counts of less than 50 × 10^3^/L (Figure 1a). An association of the total dose of Grafalon with the incidence of thrombocytopenia on POD 7 could not be observed (Figure 1b).

Similarly, the incidence of any leukocytopenia started to increase on POD 3 and peaked on POD 4, with 31% (n = 25) of patients being affected, of whom one-third displayed severe leukocytopenia (Figure 1c, Table 2). The incidence of leukocytopenia decreased to 4% (n = 3) until POD 7, all of which were mild (Figure 1c, Table 2). No case of leukocytopenia was observed on POD 14. Interestingly, a statistically significant correlation between the relative change in the number of leukocytes from POD 1 to POD 7 and the total dose of Grafalon could be observed (Spearman’s r = −0.273, *p* = 0.014, Figure 1d). Importantly, only two patients received filgrastim (one on POD 2, one on POD 3) because of critical leukocytopenia (<1 × 10^3^/L).

### 3.3. Bacterial Infections

Patients induced with Grafalon showed an incidence of severe bacterial infections during hospitalization, i.e., positive blood culture, of 21% (n = 17), all of which occurred within the first month after LT (Table 2). The rate of localized infections (i.e., pneumonia and surgical site infection) within one year after LT was 28% (n = 22, Table 2).

### 3.4. BPAR and One-Year Survival

**Figure A1:**
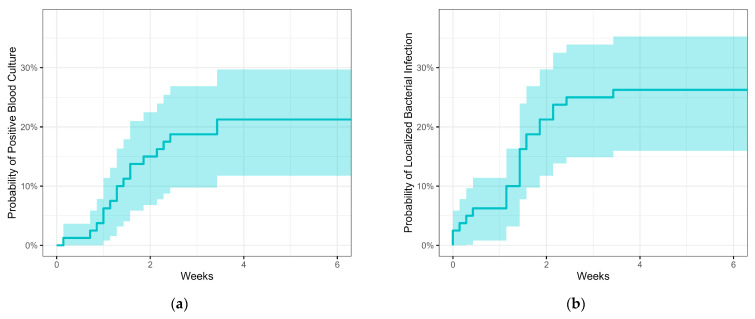

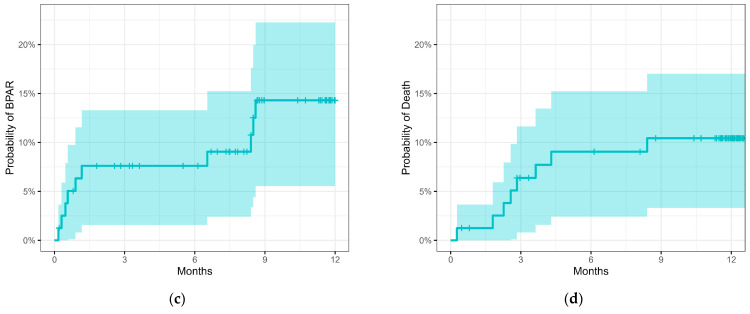
Kaplan Meier curves for positive blood culture (**a**); localized bacterial infection, i.e., pneumonia and surgical site infection (**b**); biopsy-proven acute rejection (**c**); and death (**d**). POD, postoperative day.

Overall, the rate of acute rejection within the first year after LT was 12.5% (n = 10) in the Grafalon cohort (Table 2). Interestingly, the occurrence of BPAR showed two peaks during the first postoperative year: during the first month and between six and nine months post-LT (Figure A1a). When evaluating the severity of rejection using RAI as the major scoring system, a mean RAI of 4.8 ± 1.1 could be observed. Patient survival within the first year after LT was 90% (Figure A1b). One of eight patients died from cardiac decompensation during re-transplantation following primary non-function; one patient died from multiple organ failure following chronic rejection. There was one case of acute liver failure with a fatal outcome; one patient died from a stroke. Two patients died as a consequence of infections (i.e., Enterococcus faecium sepsis and sepsis following acute or chronic kidney failure). The primary cause of death of two patients was unknown, one of which died after a fall and subdural hemorrhage. Of note, four patients (5%) were diagnosed with a solid organ tumor during follow-up, two of which were cases of HCC relapse. One patient had to undergo re-transplantation for secondary sclerosing cholangitis following an ischemic-type biliary lesion. An overview of patient outcomes and causes of death can be found in Table 2.

## 4. Discussion

We present a prospective, observational, non-interventional study evaluating the safety and efficacy of Grafalon for induction therapy in patients after liver transplantation. We found a low rate of BPAR of 12.5% in the first year after transplantation. Regarding the known bone marrow-depleting effects of induction therapy, Grafalon showed a transient decrease in leukocyte and platelet counts. Importantly, only two patients received filgrastim for critical leukocytopenia, and, after POD 7, there were no severe cases of leukocytopenia present. Furthermore, the rate of bacterial infection was found to be 49%.

The use of polyclonal antibodies like Grafalon for induction as an additional means of immunosuppression besides corticosteroids or CNIs has been established ever since the introduction of Thymoglobulin in 1984 [23]. Studies have shown that induction with Thymoglobulin reduces ischemia/reperfusion injury of liver grafts [24] and protects patients from acute rejection [25]. Another goal of induction therapy is protection from short- and long-term effects of CNI-based IS by allowing a delayed initiation or dose reduction of CNIs, resulting in reduced renal toxic effects [23].

When evaluating the primary outcome for protection from acute rejection, the rate of 12.5% BPAR in this study in the first year after LT is comparable or lower than in numerous recent reports. Bitterman et al. reported a rate of 14.9% BPAR using any T-cell-depleting agent [11], while Jorgenson et al. and Ig-Izevbekhai et al. reported 26.4% and 21.9%, respectively, using Thymoglobulin induction [26,27].

Grafalon led to a peak incidence of thrombocytopenia and any leukocytopenia on POD 4 of 64% and 31%, respectively. The latter is comparable to a recently reported rate of severe leukocytopenia after Thymoglobulin induction of 29.3% by Jorgensen et al. [26]. Furthermore, 28% of patients in the presented cohort had localized bacterial infections, while 21% of patients suffered from bacteremia; most of the infections occurred in the first postoperative month. This is comparable to Boillot et al., who, in 2009, reported a rate of antibiotically treated bacterial infections of 50% after induction with Thymoglobulin and contemporaneous CNI treatment [28]. However, the more recent report by Jorgensen et al. found a rate of any bacterial infection of 34.2% after Thymoglobulin induction, in addition to a rate of fungal infection of 15% [26].

Interestingly, in 2020, a study found potential benefits of Thymoglobulin induction in the graft survival of donation after circulatory death (DCD) liver grafts [27]. This is in line with our cohort, where all eight patients receiving DCD grafts (10%) were alive one year after LT. However, due to the small number of DCD grafts in this cohort, one cannot draw definitive conclusions concerning the effects of Grafalon induction on DCD allografts; further studies investigating this potential benefit of induction therapy are needed.

This study has several limitations. First of all, we presented a single-center, observational, non-interventional study without a paralleled control group, limiting external validity. On the other hand, the relatively large cohort comprising 80 patients and the prospective character of the data acquisition represent the strengths of this report. It has to be noted that the primary aim of this study was the characterization of Grafalon as an induction agent concerning safety and efficacy; therefore, we cannot offer clinical recommendations on the basis of these data. Furthermore, reports on induction therapy in liver transplantation are scarce; therefore, the results are difficult to compare directly. Additionally, most reports on induction therapy in LT investigate Thymoglobulin as an induction agent, limiting the external validation of the presented results.

In conclusion, Grafalon showed a rate of BPAR in the first year after LT of 12.5%, and the overall survival of this cohort was 90%. Furthermore, Grafalon led to a peak incidence of thrombocytopenia and any leukocytopenia on POD 4 of 64% and 31%, respectively. In the observed cohort, 28% of patients had localized bacterial infections, while 21% of patients suffered from bacteremia; most of the infections occurred in the first postoperative month. Five percent of patients developed post-transplant malignant diseases; of note, only two (2.5%) were de novo malignancies. Without major adverse events, Grafalon appears to be safe and, in comparison to the recent literature, effective for induction immunosuppression after liver transplantation. Further prospective studies including larger patient cohorts, a comparison of protocols with or without induction, and longer follow-up periods are needed to gain better evidence of the benefits of induction therapy.

## Figures and Tables

**Table 1:** Baseline demographics and patient data.

Characteristic	Grafalon (n = 80) ^1^
Sex (male/female)	83:17% (n = 66:14)
Age (years)	56 ± 12
BMI (kg/m^2^)	26.5 ± 4.9
MELD	15.2 ± 5.3
Na-MELD	16.9 ± 6.1
Sequelae of end-stage liver disease	
Ascites	43% (n = 34)
Hepatic encephalopathy	16% (n = 13)
Hepatorenal syndrome	13% (n = 10)
Comorbidities	
Smoking	18% (n = 14)
Hypertension	29% (n = 23)
Other cardiovascular disease	39% (n = 31)
Type 1 diabetes mellitus	2.5% (n = 2)
Type 2 diabetes mellitus	19% (n = 15)
Chronic kidney disease	16% (n = 13)
Pulmonary disease	19% (n = 15)
Primary indication for liver transplantation	
Alcoholic cirrhosis	35% (n = 28)
Virus-induced cirrhosis	2.5% (n = 2)
Hepatocellular carcinoma	30% (n = 24)
Cholestatic disease	7.5% (n = 6)
Other	25% (n = 20)
Donor type (DBD:DCD)	90:10% (n = 72:8)

^1^ Mean ± standard deviation; % (n). BMI, body mass index; MELD, model of end-stage liver disease; Na, sodium; DBD, donation after brain death; DCD, donation after circulatory death.

**Table 2:** Post-transplant outcome parameters.

Characteristic	Grafalon (n = 80) ^1^
Thrombocytopenia on POD 4	64% (n = 51)
Thrombocytopenia on POD 7	24% (n = 23)
Leukocytopenia on POD 4 (mild/severe)	20:11% (n = 16:9)
Leukocytopenia on POD 7 (mild/severe)	3.8:0% (n = 3:0)
Bacterial infection, local (i.e., pneumonia or SSI)	28% (n = 22)
Time to first local bacterial infection (weeks)	1.8 ± 2.1
Positive blood culture	21% (n = 17)
Time to first positive blood culture (weeks)	1.6 ± 0.9
Biopsy-proven acute rejection	12.5% (n = 10)
Time to biopsy-proven acute rejection (months)	3.6 ± 3.9
Post-transplant HCC recurrence	2.5% (n = 2)
De novo malignancy	2.5% (n = 2)
Death	10% (n = 8)
Time to death (months)	3.3 ± 2.4
Cause of death	
Infection	2.5% (n = 2)
Primary non-function	1.3% (n = 1)
Acute liver failure	1.3% (n = 1)
Chronic rejection	1.3% (n = 1)
Vascular disease (i.e., stroke)	1.3% (n = 1)
Unknown	2.5% (n = 2)

^1^ Mean ± standard deviation; % (n). POD, postoperative day; SSI, surgical site infection; HCC, hepatocellular carcinoma.

## Data Availability

The data presented in this study are available upon request from the corresponding author due to data privacy protection.
